# Impact of Different Foot and Mouth Disease Vaccine Schemes in Cross-Neutralization Against Heterologous Serotype O Strains in Cattle

**DOI:** 10.3390/v16111732

**Published:** 2024-11-04

**Authors:** María Cruz Miraglia, Melanie Barrios-Benito, Sabrina Galdo-Novo, Danilo Bucafusco, Ana Taffarel, Alejandra Victoria Capozzo, Manuel Victor Borca, Daniel Mariano Pérez-Filgueira

**Affiliations:** 1 Instituto de Virología e Innovaciones Tecnológicas, CICVyA, INTA-CONICET, Buenos Aires 1686, Argentina; miraglia.maria@inta.gob.ar (M.C.M.); itaffarel@senasa.gob.ar (A.T.); 2 WOAH FMD Reference Laboratory, SENASA, Buenos Aires 1640, Argentina; mebarrios@senasa.gob.ar (M.B.-B.); sgaldo@senasa.gob.ar (S.G.-N.); 3 Cátedra de Virología, Facultad de Ciencias Veterinarias, Universidad de Buenos Aires (UBA), Buenos Aires 1427, Argentina; dbucafusco@fvet.uba.ar; 4 Instituto de Investigaciones en Producción Animal, UBA-CONICET, Buenos Aires 1427, Argentina; 5 Centro de Altos Estudios en Ciencias Humanas y de la Salud, CONICET-Universidad Abierta Interamericana, Av. Montes de Oca 745, Buenos Aires 1270, Argentina; alejandravictoria.capozzo@uai.edu.ar; 6 Plum Island Animal Disease Center, ARS, USDA, Greenport, NY 11944, USA; manuel.borca@usda.gov

**Keywords:** foot-and-mouth disease, cattle, heterologous protection

## Abstract

The high antigenic variability of the foot-and-mouth disease virus (FMDV) represents a challenge for developing prophylactic strategies, stressing the need for research into vaccines offering broad protection against a range of virus strains. Here, the heterotypic cross-reaction using different vaccine schemes against serotype O strains was studied, evaluating the impact of revaccination, antigen dose, and incorporation of additional FMDV serotypes. Naïve cattle were immunized with seven distinct FMDV vaccines, receiving three doses of the same formulation at 0, 28, and 56 days post-primary vaccination (dpv). Serum samples were collected up to 70 dpv and tested by a virus-neutralizing test against serotype O strains from a South American lineage and two strains representative of two Asian lineages. Our results showed that vaccines containing the ME-SA topotype O1/Campos strain developed cross-neutralizing responses against the two Asian viruses after the first vaccination. In contrast, significant heterotypic neutralizing antibody titers against the homologous topotype strain were only found after the second vaccination, indicating that the phylogenic relationship may differ from the antigenic profiles for these two viruses. The amount of the O1/Campos strain and the revaccination were essential factors for neutralization against the homologous- and heterologous-type O FMDV viruses. The strain composition of the vaccine was only relevant for cross-neutralization against one of the Asian strains, suggesting potential intra-serotypic divergences for this pattern.

## 1. Introduction

Foot-and-mouth disease (FMD) was the first disease officially declared as notifiable by the World Organisation for Animal Health (WOAH) and remains in that category [1]. This highly contagious viral disease affects a wide range of domestic and wildlife biungulates [2] and currently remains endemic in large regions of Africa and Asia [3] with a high density of susceptible farm animals [4]. Though rarely lethal for infected animals, its main disruptive burden resides in its high morbidity rate registered among non-immunized populations. FMD outbreaks may result in substantial and long-lasting economic losses, interrupting and affecting regional and international trade in developed countries [5,6], decreasing production efficiency, and promoting loss of draught power and genetic diversity due to animal deaths in developing regions [7].

The FMD virus (FMDV) belongs to the Aphthovirus genus, Picornaviridae family [8], and infectious particles consist of a positive-sense single-stranded RNA genome within a small non-enveloped protein capsid [9]. As for other RNA viruses, the lack of proofreading or repair activities in the viral RNA polymerase [10] allows the emergence of mutations in the FMDV genome [11] and the generation of heterogeneous virus populations, which can result in a rapid emergence of antigenic variants. The significant antigenic variability among capsid proteins of different FMDV virus strains was recognized early [12] and extensively reviewed [13]. Seven FMDV serotypes have been identified based on strain restrictions to induce cross-protection in immunized animals [14]. However, limited intra-serotypic cross-reactivity has also been reported for all serotypes [15,16,17,18,19,20].

Current FMD vaccines contain chemically inactivated whole virus particles formulated in aqueous or oil adjuvants [21]. Good-quality vaccines may prevent clinical FMD [22] and transmission to other susceptible animals [23] when challenged with the homologous virus. However, vaccines must also protect against possibly circulating field strains in each region, which may differ from the vaccinal strain. Consequently, the limited cross-protection among serotypes and strains represents a major challenge in determining the appropriate formulations for vaccination campaigns [24,25]. Additionally, this problem impacts the selection of virus strains for storage in regional vaccine banks that should be deployed in the case of emergence of the disease in FMD-free zones [26]. This complexity highlights the importance of understanding the immunogenic effects of different vaccination strategies to guide vaccine formulation and strain selection.

Different approaches have been developed to select the most suitable vaccine strains based on in vitro or in vivo assays to determine cross-protective responses [27,28,29,30]. Previous results from our research group, working with serotype A FMDV strains in cattle, demonstrated that an enhancement of intra-serotypic cross-protective effects could be associated with the revaccination, the addition of extra FMDV strains, and, to a lesser extent, to the increase in the antigenic payload in the vaccine formulation [31].

Considering the potential disparity in factors affecting the cross-protective responses among FMDV serotypes, this study assesses these same variables but applied to immune responses in cattle against different heterologous FMDV strains within the serotype O. To this end, a set of immune sera was produced following different vaccination strategies and further assayed against different type O FMDV using a virus neutralization test, a serological parameter early and largely described as correlated with disease protection [32,33,34].

## 2. Materials and Methods

### 2.1. Experimental Animals

Twenty-one naive steers (200–220 kg each, 7 to 8 months old) were obtained from a livestock breeder from Chubut province, Argentina, located within the FMDV-free region, without vaccination. Animals were checked by liquid-phase blocking enzyme-linked immunosorbent assay (LPBE) for the absence of FMDV-specific antibodies upon arrival at the field of the Centro de Investigaciones en Ciencias Veterinarias y Agronómicas, Instituto Nacional de Tecnología Agropecuaria (CICVyA-INTA), and kept in these facilities during the entire experiment. All animal handling and sampling procedures were performed following biosafety and animal welfare regulations, according to protocol 18/2020 approved by the Institutional Committee for Use and Care of Experimental Animals (CICUAE) from the CICVyA-INTA.

### 2.2. Experimental Design and Sampling

Seven experimental single-oil emulsion FMD vaccines were manufactured following industrial standard procedures and further controlled and approved for experimental use by the National Agrifood Health and Quality Service (SENASA). Vaccines were formulated using inactivated FMDV 140S antigen from three different FMDV strains: O1/Campos/Brazil/58 (O1/Campos), A24/Cruzeiro/Brazil/55 (A24/Cruzeiro), and C3/Indaial/Brazil/71 (C3/Indaial). Using a 10-µg monovalent O1/Campos strain vaccine as reference (O1C 10), we formulated a second monovalent O1/Campos vaccine carrying three times that antigenic payload (O1C 30) and two additional monovalent vaccines containing 30 µg/dose of serotype A (A24/Cruzeiro, A24 30) or C strains (C3/Indaial, C3I 30). The remaining vaccines were formulated as bivalent formulations including the O1/Campos and A24/Cruzeiro strains (10 µg each, A24/O1C) or the O1/Campos and 3C/Indaial strains (10 µg each, C3I/O1C) or as a trivalent vaccine containing all three FMDV strains (10 µg each, A24/C3I/O1C) (Table 1). Vaccines were applied subcutaneously in the neck (2 mL/dose), and each experimental group (*n* = 3) received three immunizations of the same formulation at 0, 28, and 56 days post-primary vaccination. Serum samples were obtained from the jugular vein using Vacutainer^®^ (BD, Franklin Lakes, NJ, USA) tubes at 0, 7, 14, 28, 42, 56, and 70 days after the initial vaccination.

### 2.3. Assessment of Total FMDV-Specific Antibodies

Total antibodies against the O1/Campos, A24/Cruzeiro, C3/Indaial, and A/Arg/2001 (A2001) FMDV strains were measured by a liquid-phase blocking ELISA (LPBE) originally developed by Hamblin et al. [35] and further modified by Periolo et al. [36].

### 2.4. Assessment of FMDV-Specific Neutralizing Antibodies

Virus-neutralizing antibodies (nAb) against the different FMDV strains were detected in samples of bovine sera by a microplate virus neutralization test (VNT) according to recommendations of the WOAH chapter [1]. A baby hamster kidney cell line (BHK-21, clone 13) provided by INTA was used as a suspension in Dulbecco′s Modified Eagle′s (DMEM) without serum. The tests were performed at SENASA’s FMDV WOAH Reference Laboratory, consisting of two-dimensional neutralization assays, and antibody titers were calculated as the log_10_ of the reciprocal antibody dilution required for 50% neutralization of 100 tissue culture infectious doses 50% (TCID_50%_) of virus according to the Spearman–Kärber method [37].

The magnitude of the overall neutralizing performance throughout the experiment (from 0 to 70 dpv) for each experimental group against each virus strain was quantified by calculating the area under the curve (AUC) using the corresponding mean nAb time-course curves and the log_10_ of the minimum detectable nAb titer by VNT (0.7) as a baseline.

### 2.5. Virus Strains

Viral suspensions from the WOAH referent laboratory for FMDV at SENASA were assayed directly from passages in BHK-21 cells. Strains tested include FMDV vaccine strains O1/Campos, C3/Indaial, and A24/Cruzeiro and serotype O heterologous strains O/Ecuador/46/2010, O/SKR/84/YDM, and O/Taiwan/1997.

### 2.6. Indirect Reference Parameters for Assessment of the Challenge Protection

Antibody titers induced after vaccination were also referred to the “expected percentage of protection” (EPP) already established for the O1/Campos, A24/Cruzeiro, C3/Indaial, and A/Arg/01 strains for LPBE and the O1/Campos strain for VNT. The EPP estimates the likelihood that cattle would be protected after homologous FMDV challenge based on the specific antibody titers measured before challenge. EPP values referred in each case arise from correlations between the LPB-ELISA [38,39] or VNT [34] titers obtained in vaccinated cattle at 60 dpv and the in vivo challenge results obtained at 90 dpv by the “Protection against Podal Generalization” (PPG) method, involving 16 vaccinated animals infected with the homologous strain. For both total and neutralizing FMDV-specific antibodies, the EPP ≥ 75% (EPP_75_) values serve as a reference of antibody response titers associated with the protection at the population level against the homologous challenge.

The serological relationship between heterologous and vaccine (O1/Campos) strains was calculated using immune sera from cattle vaccinated with O1/Campos monovalent formulations and expressed as an r1 value resulting from the ratio between the reciprocal arithmetic mean nAb titer of immune sera against the heterologous strain, and the reciprocal arithmetic mean nAb titer of immune sera against the vaccine strain [1].

### 2.7. Statistical Analysis

Comparisons among mean AUC for whole neutralizing activity assessment and between mean VNT among experimental groups for each virus strain and time post-vaccination were carried out by one-way ANOVA followed by Tukey’s post-test for multiple comparisons (α = 0.05). Statistical evaluation of the differences in mean VNT according to the number of doses for each experimental group against the different serotype O FMDV strains was performed by a two-way ANOVA, followed by Bonferroni post-tests to compare time-points after vaccination among experimental groups. Statistical analyses were carried out using GraphPad Prism v5.0 (Prism, La Jolla, California, USA).

## 3. Results

### 3.1. Induction of Total FMDV-Specific Antibodies

**Figure 1:**
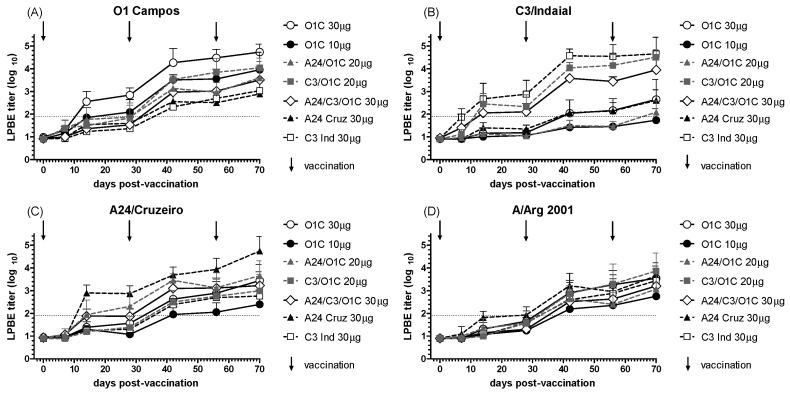
Time course of the total FMDV antibodies measured by LPBE. Seven different FMD vaccines were administered to groups of cattle (n = 3), as detailed in Table 1. Animals received three vaccinations at 0, 28, and 56 days (indicated by vertical arrows). Serum samples were taken at different times post-primary vaccination (dpv) and studied for total anti-FMDV antibodies using LPBE against the 01/Campos (**A**), C3/Indaial (**B**), A24/Cruzeiro (**C**), and A/Arg/2001 (**D**) FMDV strains. Each line depicts the mean antibody titers registered for each experimental group at the different time points + SD. Dotted lines in each chart indicate EPP_75_ corresponding to that strain by LPBE.

The immunogenicity of the experimental formulations in vaccinated cattle sera was initially analyzed by LPBE as described in the Section 2. As shown in Figure 1, all vaccination protocols induced detectable titers of FMDV-specific antibodies, which increased progressively over time. The immune response, assessed against four different FMDV strains, demonstrated that animals vaccinated with formulations containing the homologous strain—either alone, in combination with other strains, or in high-payload monovalent vaccines—developed mean antibody titers exceeding their corresponding EPP_75_ by 28 days post-vaccination (dpv) and high payload monovalent vaccines already at 14 dpv (Figure 1A–C). As expected, total antibody levels against the A/Arg/2001 strain, which was not included in the vaccines, were lower than those observed for the homologous strains (Figure 1D). Additionally, all experimental formulations resulted in increased antibody levels detected by LPBE following each revaccination, regardless of the strain tested.

### 3.2. Induction of Neutralizing FMDV-Specific Antibodies

#### 3.2.1. Antibody Responses Against the O1/Campos Strain

**Figure 2:**
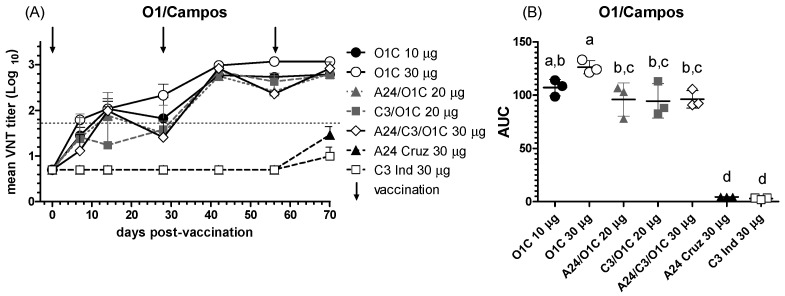
NAb responses against the O1/Campos strain. (**A**) Time course of the nAb titers in each experimental group; each line depicts mean antibody titers registered for each experimental group at the different time points. The dotted line denotes the EPP_75_ corresponding to the O1/Campos strain by VNT. Vaccination times are indicated by vertical arrows. (**B**) Overall neutralizing performance expressed as the mean AUC ± SD for each experimental group. Letters in the chart indicate significant differences between groups (1-way ANOVA *p* < 0.05; a > c, *p* < 0.01; a > d: *p* < 0.0001; b > d: *p* < 0.0001).

To test the biological activity of the immune sera, the whole set was further evaluated by VNT against different FMDV serotype O strains. Neutralizing antibody responses against the O1/Campos strain are shown in Figure 2. The *O1C* 30 μg group, receiving the highest payload monovalent O1/Campos vaccine, was the one inducing mean nAb titers above the EPP_75_ value for VNT (1.65) already at 7 dpv and maintaining an increasing trend all along the experiment (Figure 2A). To estimate the overall neutralizing performance across the experimental groups throughout the trial, the area under the curve (AUC) was calculated and compared among them. As shown in Figure 2B, cattle from the *O1C* 30 μg group presented a significantly higher neutralizing activity than the remaining experimental groups, except the *O1C* 10 μg group. However, neutralizing activity in animals vaccinated with formulations combining other serotypes (*C3I/O1C* 20 μg, *A24/O1C* 20 μg, and *A24/C3I/O1C* 30 μg groups) were similar to those of the O1C 10 μg group. Monovalent vaccines from the A and C serotypes only induced detectable levels of nAb after the third vaccine dose (Figure 2A).

#### 3.2.2. Antibody Responses Against Heterologous Serotype O South American Strains

**Figure 3:**
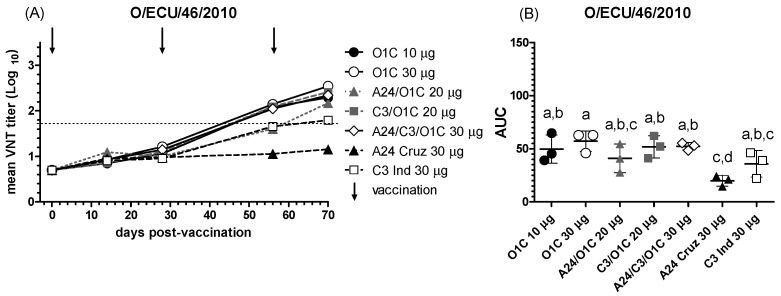
NAb responses against the O/ECU/46/2010 strain. (**A**) Time course of the nAb titers in each experimental group. Each line depicts mean antibody titers registered for each experimental group at the different time points against the O/ECU/46/2010 strain. The dotted line denotes the EPP_75_ corresponding to the O1/Campos strain by VNT. Vaccination times are indicated by vertical arrows. **(B**) Overall neutralizing performance expressed as the mean AUC ± SD for each experimental group against the O/ECU/46/2010 strain. Letters in the chart indicate significant differences between groups (1-way ANOVA *p* < 0.05; a > d: *p* < 0.001; b > d: *p* > 0.01).

Next, the heterologous serotype O strain O/ECU/46/2010, which belongs to the same topotype as the O1/Campos strain and was isolated during the FMDV outbreaks in Ecuador (2009–2011) [40], was evaluated against the set of immune sera (Figure 3). As shown in Figure 3A, the O/ECU/46/2010 strain was significantly neutralized only after the second vaccination by sera from cattle vaccinated with formulations containing the O1/Campos strain, either alone or in combination with the A24/Cruzeiro or C3/Indaial strains (in bivalent or trivalent formulations). In contrast, sera from animals vaccinated with the high-payload A24/Cruzeiro monovalent vaccine exhibited almost no neutralizing activity throughout the sampling period, with overall neutralizing performance significantly lower than that of most vaccines containing the O1/Campos strain (Figure 3B).

#### 3.2.3. Antibody Responses Against the Heterologous Serotype O Asian Strains

**Figure 4:**
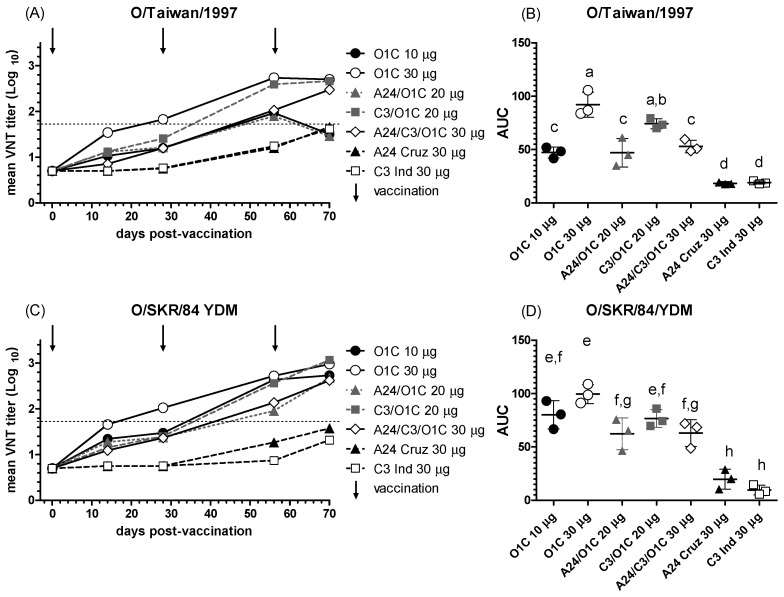
NAb responses against the O/Taiwan/1997 and O/SKR/84/YDM strains. (**A**,**C**) Time course of nAb titers in each experimental group. Each line depicts mean antibody titers registered for each experimental group at the different time points against the O/Taiwan/1997 (**A**) or O/SKR/84/YDM (**C**) strains. Dotted lines denote the EPP_75_ corresponding to the O1/Campos strain by VNT. Vaccination times are indicated by vertical arrows. (**B**,**D**) Overall neutralizing performance expressed as the mean AUC ± SD for each experimental group against the O/Taiwan/1997 (**B**) or O/SKR/84/YDM (**D**) strains. Letters in the chart indicate significant differences between groups (1-way ANOVA *p* < 0.05; a > c, *p* < 0.0001; a > d, *p* < 0.0001; b > c, *p* < 0.001; c > d: *p* < 0.01; e > g, 0.001; e > h, 0.0001; g > h, 0.001).

Immune sera from animals vaccinated with the experimental formulations were further tested against two additional serotype O strains: O/SKR/84/YDM and O/Taiwan/1997, belonging to the topotypes SEA Lineage MYA-98 and Cathay, respectively. Neutralizing responses against Asian FMDV strains tested showed different patterns of reactivity compared to the heterologous South American strain. Significant neutralizing activity against both strains was already detected in sera from cattle vaccinated with the high payload monovalent O1/Campos vaccine (*O1C* 30 μg) two weeks after the first vaccination, with mean neutralizing titers ≥ 1.5 (Figure 4A,C). Also, for both strains, sera from animals immunized with the C3I/O1C bivalent vaccine showed VNT titers above 2.0 at 56 dpv after the first revaccination (Figure 4A,C).

Sera from steers vaccinated with the other bivalent formulation (*A24/O1C* 20 μg) also showed neutralizing activity against both heterologous strains (Figure 4A,C) but in lower levels than in the *C3I/O1C* 20 μg group, reaching similar VNT titers at 70 dpv and only for the O/SKR/84/YDM strain (Figure 4C). The trivalent formulation also induced increasing VNT titers over time, reaching values > 2.0 after the first revaccination (56 dpv), but in lower levels than the monovalent formulation carrying the same antigenic payload (O1/Campos 30 μg/dose). Only at 70 dpv were VNT titers induced against both heterologous strains similar between the trivalent and high-payload monovalent O1/Campos vaccines. Both monovalent vaccines formulated with A or C serotype strains only showed detectable VNT titers against these O serotype heterologous strains after the second revaccination at 70 dpv.

Considering the overall mean neutralizing performance against the O/Taiwan/1997 strain, the higher-payload O1/Campos monovalent vaccine group (*O1C* 30 µg) was above all the remaining groups, except for the *C3I/O1C* 20 μg (Figure 4B). All vaccines containing the O1/Campos strain elicited neutralizing activities significantly above those of the A24/Cruzeiro and C3/Indaial monovalent formulations (Figure 4B). The *O1C* 30 µg group also showed higher mean overall neutralizing effect against the O/SKR/84/YDM strain than the *A24/O1C* 20 μg, *A24/C3I/O1C* 30 μg, *A24* 30 μg, and *C3I* 30 μg groups (Figure 4D). Performance of the *O1C* 10 µg and *C3I/O1C* 20 μg groups showed non-significant differences against both the *O1C* 30 µg group and the *A24/O1C* 20 μg and *A24/C3I/O1C* 30 μg groups. As described for the O/Taiwan/1997 strain, all vaccines containing the O1/Campos strain elicited mean nAb performances significantly above those of the A24/Cruzeiro and C3/Indaial monovalent formulations (Figure 4D).

### 3.3. Effect of the Vaccine Schedule and Strain Composition in the Neutralizing Activity of the Immune Sera

#### 3.3.1. Vaccination Schedule

**Figure 5:**
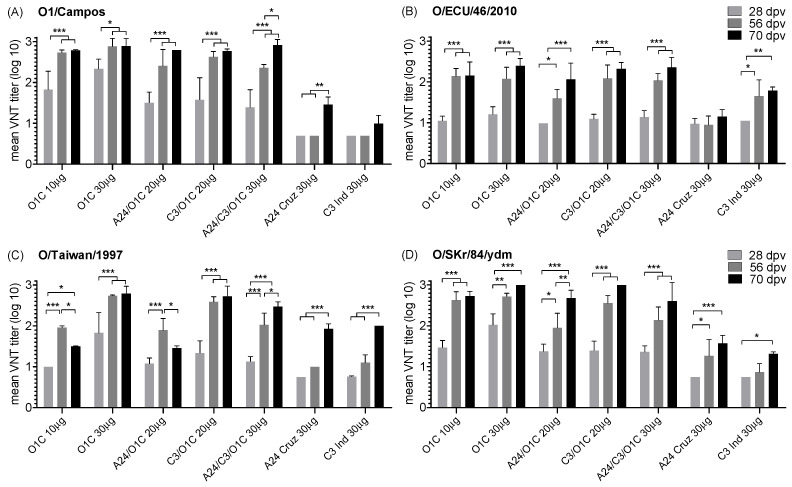
Effect of number of doses in the neutralizing activity of the immune sera against different serotype O FMDV strains. Bars indicate mean VNT titers obtained for each experimental group (n = 3) ± SD after the first (28 dpv), second (56 dpv), and third vaccination (70 dpv). Antibodies were measured against the O1/Campos (**A**), O/ECU/46/2010 (**B**), O/Taiwan/1997 (**C**), or O/SKR/84/YDM (**D**) FMDV strains. Data sets were analyzed by 2-way ANOVA, and significant differences are indicated as asterisks (* *p* < 0.05, ** *p* < 0.01 and *** *p* < 0.001).

The effect of the number of doses of the experimental formulations in the nAb responses elicited was evaluated by comparing the mean VNT titers measured after the first (28 dpv), second (56 dpv), and third vaccination (70 dpv) against the homologous and heterologous serotype O FMDV strains. As a general observation, revaccinations improved the neutralizing capacity in all the experimental formulations against all FMDV strains tested (Figure 5). Significant differences among immunizations were often found between the first and second vaccination in formulations containing the O1/Campos strain. This was observed for all formulations against the O/ECU/46/2010 and O/SKR/84/YDM (Figure 5B,D), four out of the five formulations against the homologous strain (Figure 5A), and three out of the five against the O/Taiwan/1997 strain (Figure 5C).

#### 3.3.2. Vaccine Formulation

The progression of mean nAb titers induced by each experimental vaccine against the homologous and heterologous serotype O FMDV strains was analyzed two and four weeks after the first vaccination (14 dpv and 28 dpv, respectively), four weeks after the second vaccination (56 dpv), and two weeks after the third vaccination (70 dpv).

**Figure 6:**
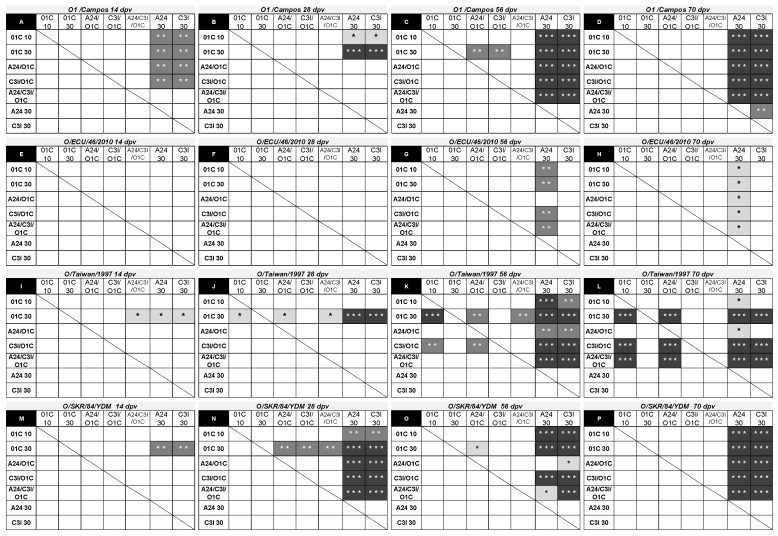
Effect of the formulation in the neutralizing activity of the immune sera against different serotype O FMDV strains. (**A**–**P**) Each chart shows significant differences existing in the induced mean VNT titers between each formulation in the first column (in bold) compared with the rest of the formulations indicated in the first row. Corresponding virus strains and post-vaccination time points are indicated at the top of each graph. Significant differences are indicated in shaded boxes, and the significance level is denoted as asterisks (1-way ANOVA; * *p* < 0.05, ** *p* < 0.01 and *** *p* < 0.001).

At 14 dpv, nAb responses against the homologous strain were already significantly higher than those induced by the A24/cruzeiro and C3/Indaial monovalent vaccines in four out of five of the vaccines containing the FMDV O1/Campos (Figure 6A). At 28 dpv, significant differences were limited to both monovalent O1/Campos vaccine groups (Figure 6B). After the second vaccination, all vaccine groups containing the O1/Campos strain were significantly different than the *A24* 30 μg and *C3I* 30 μg groups (Figure 6C,D), while significant differences between the mean VNT titers elicited by the O1/Campos 30 μg vaccine and both bivalent vaccines were transiently observed only at this time (56 dpv) (Figure 6C). The *O1C* 30 μg vaccine group, although increasing the VNT titers induced over time, did not show significant differences from cattle immunized with the lower monovalent O1/Campos or the trivalent vaccines in all tested time-points (Figure 6A–D).

Humoral immune neutralizing responses against the South American heterologous strain O/ECU/46/2010 were not different among vaccines after the first vaccination. Neutralizing antibody responses induced by vaccines containing the O1/Campos strain showed significant differences only after the second vaccination (56 dpv) and were restricted to the *A24* 30 μg vaccine group (Figure 6G,H).

In the case of the O/Taiwan/1997 strain, significant differences in nAb responses at 14 dpv were limited to the *O1C* 30 μg group against of the *A24* 30 μg, *C3I* 30 μg, and *A24/C3/O1C* 30 μg groups (Figure 6I). The progression of the responses was also different from that observed against the homologous strain: differences increased their significance and were extended to the rest of the experimental groups at 28 dpv, except against the *C3I/O1C* 20 μg vaccine group. This last group also presented nAb titers above those of the *A24* 30 μg and *C3I* 30 μg groups (Figure 6J). After the second vaccination (56 dpv), nAb titers in the *A24* 30 μg or *C3/I* 30 μg groups were lower than the rest of the animals vaccinated with formulations containing the O1/Campos strain, with increased significance. Additionally, the *O1C* 30 μg and *C3I/O1C* 20 μg groups presented significantly higher nAb titers than the *A24/O1C* 20 μg and *A24/C3/O1C* 30 μg groups (Figure 6K). After the third vaccination, the *O1C* 30 μg, *C3I/O1C* 20 μg, and *A24/C3/O1C* 30 μg groups were similar among them and significantly higher than the rest of the groups. Contrarily, significant differences between the *O1C* 10 μg and *A24/O1C* 20 μg groups against the *C3I* 30 μg group disappeared (Figure 6L).

Neutralizing responses against the O/SKR/84/YDM strain at 14 dpv were only significantly higher in the *O1C* 30 μg group compared to its counterparts from another serotypes (*A24* 30 μg and *C3I* 30 μg) (Figure 6M). As observed for the other Asian strain, two weeks later (28 dpv), these differences increased their significance, were extended to the rest of the experimental groups, and were also detected for all animals immunized with bivalent and trivalent vaccines against the *A24* 30 μg and *C3I* 30 μg groups (Figure 6N). After revaccination (56 dpv), most of the significant differences against the *A24* 30 μg and *C3I* 30 μg groups remained, in some cases increasing their significance, and the nAb levels induced in the *O1C* 30 μg group were reached by the *O1C* 10 μg, *C3I/O1C* 20 μg, and *A24/C3/O1C* 30 μg groups (Figure 6O). After the third vaccination (70 dpv), all vaccines containing the O1/Campos strain induced similar nAb titers among them and were significantly higher than those observed for the *A24* 30 μg and *C3I* 30 μg groups, resembling the results observed against the homologous FMDV strain (Figure 6P,D).

### 3.4. Serological Relationship Between a Heterologous Strains and Vaccine Virus

Following the guidelines of the WOAH [1], a one-way serological relationship index (r1) was calculated for each heterologous O serotype strain using the immune sera from cattle vaccinated with the monovalent O1/Campos formulations (10 and 30 μg/dose) at 14 and 28 dpv following primary vaccination.

As detailed in Table 2, the r1 values for each heterologous strain varied depending on the antigenic payload of the O1/Campos vaccine and the time post-vaccination considered. Given the accepted index threshold for VNT that assigns a match between a vaccine and heterologous strain (r1 ≥ 0.3) and also the need for a vaccine inducing robust homologous nAb responses [41], only cross-neutralizing responses induced by the higher payload O1/Campos vaccine against the Asian strains met both criteria.

## 4. Discussion

A previous report from our research group investigated the effects of antigenic payload, strain composition, and number of doses on the heterologous protection in cattle between two FMDV serotype A strains, A24/Cruzeiro and A/Arg/2001, which, as earlier described, showed limited cross-protective responses between them [20,42]. Based on its vast global distribution [3] and since the relevance of vaccination protocols influencing cross-protective responses may differ among different FMDV serotypes, this study examines these same variables but related to immune responses against different heterologous FMDV strains within serotype O.

Our findings with FMDV type A provided clear evidence that enhanced intra-serotypic cross-protective effect could be achieved by combining the A24/Cruzeiro strain with others from different serotypes (O1/Campos and C3/Indaial) or after revaccination with a regular payload A24/Cruzeiro monovalent vaccine (10 μg/dose). Although increasing payload up to four times (40 μg/dose) in a monovalent A24/Cruzeiro vaccine also improved heterologous protection, only the trivalent formulation (containing A24/Cruzeiro, O1/Campos, and C3/Indaial strains) and revaccination with the lower payload monovalent A24/Cruzeiro vaccine could afford 100% protection against the in vivo heterologous challenge with the FMDV A/Arg/2001 strain in cattle [31].

To determine if these trends also apply to strains within serotype O, a group of naïve cattle were vaccinated with seven different oil-based inactivated FMDV vaccines, which varied in strain compositions and antigen payload (see Table 1). Each group of cattle received three doses of the same vaccine formulation at 0, 28, and 56 days after the initial vaccination. This approach allowed assessment of the effect of revaccination, antigen dose, and the incorporation of additional FMDV serotypes on the immune sera’s ability to neutralize three different heterologous serotype O FMDV strains. The homologous O1/Campos vaccine strain was used as a reference, and a virus neutralization test (VNT) assay was conducted to evaluate cross-neutralizing activity.

As expected, all experimental vaccines triggered the production of FMDV-specific antibodies in naïve cattle. The levels of total FMDV-specific antibodies, assayed using LPBE, increased throughout the experiment, particularly following booster vaccinations. The humoral responses, tested against four different FMDV types, exhibited a clear strain-specific pattern, with higher antibody titers observed when the vaccines contained the same strain as the one used in the LPBE tests. Homologous immune responses against the O1/Campos, A24/Cruzeiro, and C3/Indaial strains already surpassed the EPP_75_ threshold for the corresponding strain at 14 dpv, demonstrating their immunogenic ability. Furthermore, as expected, specific antibody responses against the A/Arg/01 strain, not present in any of the experimental formulations, were lower than for the rest of the vaccinal strains and were only higher than its corresponding EPP_75_ after the second vaccination.

The neutralizing activity of the set of immune sera against the O1/Campos strain heavily depended on including the homologous virus in the experimental vaccines. As opposed to the results with serotype A strains in cattle [31], the inclusion of heterologous serotype strains did not improve the performance of the lower-payload monovalent O1/Campos vaccine (10 μg/dose) at any of the tested times or in the overall neutralizing activity along the experiment. Revaccination significantly increased the VNT titers against the O1/Campos strain in all the experimental groups, except for the *C3I* 30 μg group, which practically did not show neutralizing responses during the experiment. Interestingly, for most of the vaccines containing the O1/Campos strain, significant increases in homologous VNT titers were registered between the first and second vaccination but not between the second and third vaccination. After the third vaccination, all the vaccines containing the O1/Campos strain reached similar mean nAb titers.

Evidence of nAb responses against the South American heterologous strain O/ECU/46/2010 was only observed after the second vaccination for all the formulations, except for the group immunized with the serotype A monovalent vaccine (*A24* 30 μg), which remained negative for strain-specific nAb throughout the experiment. Despite their phylogenetic proximity to the O1/Campos strain [40], their antigenic differences resulted in a significant decrease in the neutralizing activity for all experimental vaccines compared to their performance against the homologous strain. The serological results presented here are in line with previous in vivo challenge experiments, which showed that a 20 μg monovalent O1/Campos vaccine only provided 6.25% of protection against the O/ECU/46/2010 strain challenge in PPG tests after a single vaccination and 18.75% after revaccination [43]. As with the O1/Campos strain, revaccination significantly increased the neutralizing activity against the O/ECU/46/2010 strain for most of the vaccine groups, including the *C3I* 30 μg group, especially between the first and second vaccination, though to a lesser extent. However, the highest mean neutralizing titers achieved with vaccines containing the O1/Campos strain were nearly ten times lower than those observed in the neutralization tests with the homologous virus. Additionally, consistent with the VNT results obtained against the O1/Campos strain, the inclusion of heterologous strains did not enhance the performance of the lower payload monovalent O1/Campos vaccine (10 μg/dose). Two weeks after the third vaccination (70 dpv), all these vaccines reached similar titers, with significant differences observed only against the monovalent A24/Cruzeiro vaccine (30 μg/dose).

Unlike the South American strain, both Asian type O FMDV viruses showed significant cross-neutralization with the immune sera from cattle vaccinated with the higher-payload monovalent O1/Campos vaccine (30 μg/dose) after primary vaccination. Mean nAb titers were 1.54 (14 dpv) and 1.83 (28 dpv) for the O/Taiwan/1997 strain and 1.66 (14 dpv) and 2.02 (28 dpv) for the O/SKR/84/YDM strain. All these values are above the suggested cut-off titer for VNT (1.5) previously proposed as an indicator of heterologous cross-protection using serum samples collected 21 dpv [28]. In line with this, our results showed that estimation of the serological relationship index (r1), as described by the WOAH manual [1], was also indicative of a significant cross-neutralization of the O/Taiwan/1997 and O/SKR/84/YDM strains, with immune sera from the higher monovalent O1/Campos vaccine already present at 14 dpv, being the opposite of the O/ECU/46/2010 strain, even at 28 days post-primary vaccination. These results also concur with previous r1 estimations performed for these two Asian strains using sera from cattle immunized with an FMDV O1/Campos monovalent vaccine and collected at 27 dpv [44]. Interestingly, the O/SKR/84/YDM and O/Taiwan/1997 strains correspond to topotypes SEA Lineage MYA-98 and Cathay, respectively, which are phylogenetically distant to the vaccinal O1/Campos strain [45]. As observed for the O/ECU/46/2010 strain, this may indicate that the phylogenetic classification provides different information from that of the corresponding antigenic profiles for these viruses. As mentioned for the O1/Campos and O/ECU/46/2010 strains, the increase in the O1/Campos strain payload and the revaccination were essential factors in the neutralizing responses against both Asian FMDV viruses. However, cross-neutralizing responses against the FMDV O/Taiwan/1997 induced in the *O1C* 10 μg group were also improved by adding the C3/Indaial strain after the second vaccination and in the trivalent formulation after the third vaccination, indicating that the strain composition of the vaccine may also be relevant for cross-neutralization against this virus.

In summary, this study highlights that the antigenic payload of the homologous serotype vaccine strain is essential for generating cross-neutralizing responses among the tested serotype O strains. Consistent with our findings for serotype A strains [31], revaccination was a significant factor in achieving intra-serotypic cross-neutralization for type O strains. For most vaccine formulations and strains evaluated, the first revaccination typically triggered stronger recall-neutralizing responses than the second booster vaccination.

Formulations combining heterologous serotype strains were less impactful than in the previous serotype A trials, with significance observed only for the O/Taiwan/1997 virus with formulations including the C3/Indaial strain, suggesting potential differences in the relevance of this feature among strains within the same serotype. Lastly, our results suggest that genomic information, at least based on VP1 phylogenetic trees for the FMDV type O viruses tested, may not correspond with intra-serotypic cross-neutralizing antigenic profiles. Mapping the antigenic regions involved in cross-neutralization could provide valuable insights into this issue.

These results indicate that antigenic bases of potential cross protection among heterologous strains within a particular FMDV serotype could vary among the serotype considered. The possible rationale explaining this differential behavior among serotypes is, at this point, uncertain.

## Figures and Tables

**Table 1:** Strain composition of experimental FMD vaccines. Seven single oil-emulsion vaccines were formulated using three different inactivated FMDV strains (O1/Campos, A24/Cruzeiro, and C3/Indaial). The amount of antigen in each vaccine is expressed as µg of inactivated FMDV 140S particles per dose. Each vaccine (2 mL/dose) was administered in the corresponding experimental groups (n = 3) at 0, 28, and 56 days post-primary vaccination.

Experimental Groups	FMDV Strains (µg/Dose)		
	O1/Campos	A24/Cruzeiro	C3/Indaial
*01C* 10 μg	10	-	-
*01C* 30 μg	30	-	-
*A24* 30 µg	-	30	-
*C3I* 30 μg	-	-	30
*A24/O1C* 20 μg	10	10	-
*C3I/O1C* 20 μg	10	-	10
*A24/C3I/O1C* 30 μg	10	10	10

**Table 2:** Serological relationship index values for heterologous serotype O strains. Serological relationship indexes (r1) were determined using mean VNT titers from experimental groups immunized with the 10 or 30 μg/dose monovalent O1/Campos vaccines against the homologous and the indicated heterologous serotype O strains at 14 and 28 days post-primary vaccination. Numbers in bold indicate r1 ≥ 0.30.

Experimental Groups	Heterologous FMDV Strains					
	O/ECU/46/2010		O/SKR/84/YDM		O/Taiwan/1997	
	14 dpv	28 dpv	14 dpv	28 dpv	14 dpv	28 dpv
*01C* 10 μg	0.07	0.11	0.20	0.28	0.10	0.15
*01C* 30 μg	0.08	0.15	**0.42**	**0.49**	**0.32**	**0.31**

## Data Availability

Data are contained within the article.
